# Male aging in germ cells: What are we inheriting?

**DOI:** 10.1590/1678-4685-GMB-2024-0052

**Published:** 2025-02-18

**Authors:** Arturo Elías-Llumbet, Sebastián Lira, Marcia Manterola

**Affiliations:** 1 University of Groningen, University Medical Center Groningen, Department of Biomedical Engineering, Groningen, Netherlands.; 2 University of Chile, Faculty of Medicine, Institute of Biomedical Sciences, Human Genetics Program, Santiago, Chile.; 3 Universidad Andres Bello, Research Center for Sustainability, Santiago, Santiago, Chile.; 4 University of Valparaíso, Center for Translational Studies in Stress and Mental Health (C-ESTRES), Valparaíso, Chile.

**Keywords:** Germ cells, aging, mutations, epigenetics, fertility

## Abstract

Aging is a significant risk factor for male fertility and can lead to severe developmental disorders in offspring. It disrupts testicular function and spermatogenesis, resulting in sperm abnormalities and DNA fragmentation. Male aging alters the genome and epigenome of germ cells due to persistent oxidative stress caused by the cumulative effects of environmental factors over a lifetime. At the molecular level, DNA damage occurs and is poorly repaired due to impaired DNA repair pathways, leading to unrepaired lesions and *de novo* mutations. Aging also creates distinct epigenetic landscapes that modify gene expression in germ cells, affect the DNA damage response, and generate *de novo* DNA and epigenetic mutations that are transmitted to the sperm and inherited by the offspring. This review discusses current knowledge on the age-associated effects on male germ cells and the genomic and epigenomic mechanisms contributing to altered male reproductive health and outcomes in progeny. We propose a male reproductive aging threshold, where cumulative exposure to risk factors leads to oxidative stress, impaired spermatogenesis, and altered reproductive outcomes. Finally, we discuss novel interventions to prevent premature testicular aging and emphasize the need for public health policies and counseling guidelines for men seeking paternity.

## Introduction

For many years, reproductive aging has been considered primarily a women’s issue. However, increasing evidence shows that paternal age has significant consequences on reproductive health and the risk of disease in offspring. Over the last 30 years, the average age at which men are having children has increased dramatically. Although most statistical data come from maternal records, at least in developed countries, the average age of fatherhood has risen by 3.5 years, reaching an average of 30.9 years at the time of their child’s birth (Kovac et al., 2013; Khandwala et al., 2017).

In South America, data on paternal demographics are still limited and primarily focused on mothers. In Chile, the majority of births shifted from fathers aged 25-29 years in 2010 to those aged 30-35 years in 2019 (INE, 2017-2019). Moreover, the percentage of fathers aged 30-44 years increased from 41.4% to 50.3% in less than ten years (INE, 2017-2019), indicating that most births in Chile are currently from fathers aged 30 years and older.

Although the implications of delayed fatherhood are often understudied due to the lifelong production of sperm, advanced paternal age (APA) at conception (≥40 years) has become a public health issue. Older fathers exhibit reproductive deficiencies and have a significant impact on the health of their progeny (Liu et al., 2011; Eisenberg and Meldrum, 2017; Janecka et al., 2017; De Jonge and Barratt, 2019; Chan and Robaire, 2022).

## Age influences male reproductive function 

**Figure 1-:**
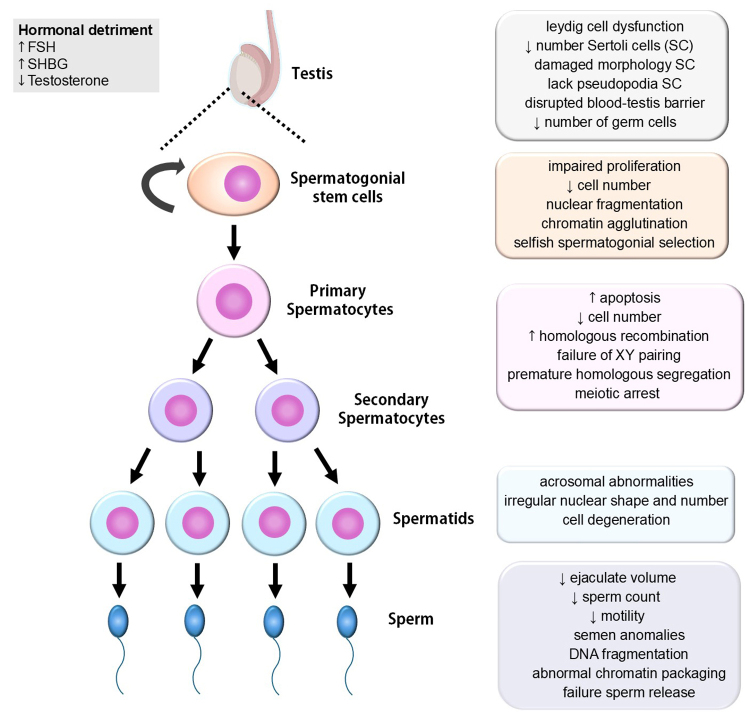
The effects of aging on male reproductive function, hormonal balance, and spermatogenesis. The boxes highlight the specific detrimental impacts of aging on each stage of germ cell development.

The effect of APA on reproductive parameters has been well described (Gunes et al., 2016; Laurentino et al., 2020). Several cross-sectional studies of healthy men have found that testicular volume changes with age due to impaired spermatogenesis (Lasiene et al., 2021; Matzkin et al., 2021; Dong et al., 2022). Significant changes are also observed in ejaculate volume, sperm count and motility, semen anomalies, DNA fragmentation in sperm, and increased time-to-pregnancy, among others (Figure 1) (Johnson et al., 2015; Halvaei et al., 2020; Laurentino *et al*., 2020; Dong *et al*., 2022), leading to reduced fertility. Men over 40 show increased time to conceive (Ford et al., 2000) and lower efficacy after undergoing assisted reproductive technologies (Belloc et al., 2008; Girsh et al., 2008). These reproductive changes, associated with normal male reproductive aging, have also been observed in species ranging from *C*. *elegans* (Tatar 2010) and fruit flies (Sepil et al. 2020), to birds (Ottinger, 2010) and mammals, including rodents (Koeva et al., 2009; Sepil *et al*., 2020; Han et al., 2021), ferret (Wolf et al., 2000), bull, ram, buck, boar, dog, and stallion (for a review, see (Comizzoli and Ottinger, 2021; Abah et al., 2023).

Regarding endocrine parameters, male aging slightly increases follicle-stimulating hormone (FSH) and sex hormone-binding globulin (SHBG), which are associated with a decrease in free testosterone after the age of 40 (Figure 1) (Laurentino et al., 2020). This is linked to changes in hypothalamic-pituitary-testicular axis function (Veldhuis et al., 2009) and Leydig cell dysfunction in elderly men (Mularoni et al., 2020). However, recent data indicate that the impact of hormone production on testicular function deterioration is less significant than the specific processes occurring in germ cells during aging (for a review, see Pohl et al., 2021).

The support, protection, and nutrition of spermatogenic cells provided by Sertoli and Leydig cells are also altered with age. Sertoli cells exhibit lower numbers and damaged morphology and function. In rats, Sertoli cells lack the pseudopodia necessary for spermatid elongation and residual body phagocytosis, leading to the cessation of spermatogenesis in some tubules or parts of the seminiferous tubule (Humphreys 1977) and disrupting the blood-testis barrier (Figure 1) (Santiago et al., 2019). In mice, aged testes show ~20% of seminiferous tubules with germ cell depletion, disrupted germ cell associations, and failure in sperm release, while the remaining 80% show a decreased number of germ cells and average reduced proliferation of spermatogonia (Figure 1) (Endo et al., 2024). 

## Aging effects in spermatogenesis

Germ cell loss and impaired spermatogenesis are key characteristics of male aging. Spermatogenesis, the process by which sperm cells are produced, occurs in the seminiferous tubules of the testes. This complex, highly regulated process begins with spermatogonia, diploid germ cells (2n) that undergo mitotic divisions. Spermatogonial stem cells (SSCs) are further divided into A-spermatogonia, which do not undergo mitosis and remain as stem cells (AdVac), and B-spermatogonia, which enter mitosis to differentiate and proliferate into spermatocytes.

In the meiotic phase, spermatocytes undergo a specialized form of cell division involving a single round of DNA replication followed by two rounds of chromosome segregation, producing four genetically distinct haploid spermatids from one parent cell. During spermiogenesis, these spermatids undergo significant morphological changes to mature into genetically and epigenetically competent sperm.

**Figure 2-:**
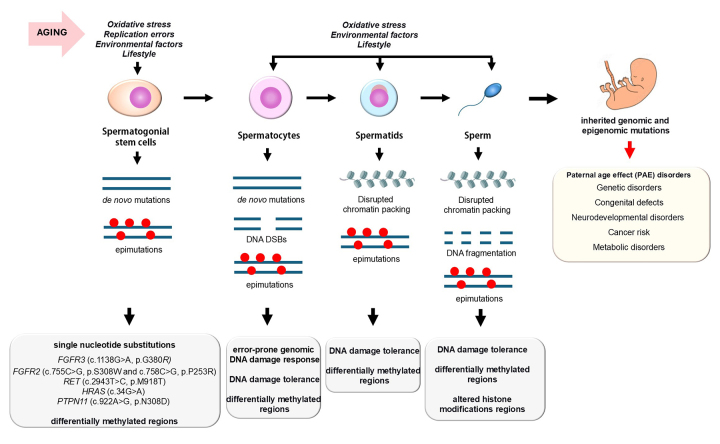
Genomic and epigenetic alterations in germ cells and sperm that occur during male aging can lead to the inheritance of diseases in offspring. Aging effects are further accelerated and exacerbated by environmental factors, lifestyle choices, and oxidative stress. Each type of genetic and epigenetic defect is produced in specific cell types, and when these defects are not repaired, they can be passed on to spermatocytes, spermatids, and sperm. The nature of these mutations is cell-type specific, as indicated in the grey boxes. These genomic and epigenomic mutations may then be inherited by the embryo, leading to reprogramming of the offspring’s genome, which can influence their phenotype and contribute to the development of various disorders and diseases (light yellow box).

With aging, SSCs, spermatocytes, spermatids, and sperm are impacted by an imbalance of proliferation and differentiation factors, increased free radical damage, abnormal metabolism, impaired DNA repair, DNA damage, and the accumulation of *de novo* mutations (Figure 2). Male aging is associated with impaired SSC proliferation, a reduction in spermatogonial numbers, nuclear fragmentation, and chromatin agglutination (Figure 1) (Nistal et al., 1987; Endo et al., 2024). Interestingly, with advancing age, the proliferation rates of AdVac spermatogonia specifically increase, a phenomenon referred to as spermatogenic efficiency. This compensatory proliferation, however, compromises the integrity of SSCs. (Pohl et al., 2019). The continuous cell divisions and repeated rounds of DNA replication in spermatogonia increase the risk of replication errors and genomic instability. In this context, the accumulation of *de novo* mutations enables clonal expansion of mutant spermatogonia over time, resulting in what is termed selfish spermatogonial selection (Goriely and Wilkie, 2012). For instance, *de novo* mutations in key genes within the tyrosine kinase pathway, such as fibroblast growth factor receptor 2 (FGFR2) or its paralog FGFR3 promote the proliferative expansion of mutant cells at the expense of normal spermatogonia (Figure 2) (Maher et al., 2016). Additionally, with aging, fibroblast growth factors (FGFs) progressively disrupt the homeostatic regulation of SSCs, leading to the loss of quiescence (Pohl *et al*., 2021).

Spermatocytes are significantly affected by male aging, displaying increased apoptosis, reduced cell numbers, and various morphological changes (Figure 1) (Barnes et al., 1998). Since meiotic spermatocytes undergo chromosome synapsis, DNA recombination, high levels of transcription, and chromosome segregation, any errors during these processes can result in severe consequences for the resulting gametes. For instance, in mice, aging is associated with increased recombination between homologous chromosomes, failure of XY chromosome pairing, and premature separation of autosomal homologs during metaphase I (Zelazowski *et al*., 2017). Additionally, male aging leads to elevated levels of O-linked N-acetylglucosamine (O-GlcNAc), which is linked to meiotic arrest at the pachytene stage due to defects in chromosome synapsis and recombination (Qian et al., 2023). Despite these defects during prophase I, no evidence of aneuploidy is observed at metaphase II, suggesting that spermatocytes with chromosomal abnormalities are eliminated during meiosis in aged mice (Vrooman et al., 2014).

In spermatids, aging is associated with acrosomal abnormalities, the presence of cytoplasmic droplets, irregular nuclear shapes, and even the appearance of multinucleated spermatids within the seminiferous tubules, ultimately leading to cell degeneration (Nistal et al., 1986; Paoli et al., 2019; Fice and Robaire, 2023; Kleshchev et al., 2023). Aged sperm also exhibit altered morphology, with disrupted flagella, nuclear membrane irregularities, and changes in mitochondrial content (Figure 1) (Calvo et al., 1995).

## Aging effects in sperms and epididymis

After completing maturation, spermatids develop into sperm, which are released into the tubular lumen in a process called spermiation. Sperm maturation continues in the epididymis, where they interact with the luminal environment of each epididymal region. This interaction influences sperm concentration, the acquisition of motility, fertilization ability, and storage. Epididymal cells communicate with sperm via epididymal-derived exosomes (epididymosomes), which play a role in the epigenetic inheritance of paternal traits (James et al., 2020). From Drosophila to humans, age-related alterations in sperm are observed, impacting sperm count, morphology, function, and motility (Lopez-Otin et al., 2013; Johnson et al., 2015; Halvaei et al., 2020; Sepil et al., 2020). In humans, daily sperm production decreases by more than 30% in men over 50 (Neaves et al., 1984). Other studies indicate a reduction in sperm concentration by approximately 3.3% per year of age, and a decrease in sperm motility by 1.2% every five years (Auger et al., 1995). Furthermore, the frequency of sperm with coiled and short tails, as well as amorphous, elongated, or vacuolated heads, increases in men over 40 (Kleshchev et al., 2023).

A hallmark of reproductive aging is the decline in sperm DNA integrity, characterized by increased DNA fragmentation and abnormal chromatin packaging (Figure 2) (Ozkosem et al., 2015; Gonzalez et al., 2021; Szabo et al., 2023). Men over 40 exhibit 1.65 times higher levels of sperm DNA fragmentation compared to those under 40 (Kaarouch et al., 2018; Lahimer et al., 2023). While some studies report that aging affects sperm DNA decondensation, others do not find significant changes (Kaarouch *et al*., 2018; Lahimer *et al*., 2023). Nevertheless, increased sperm DNA damage is more prevalent in APA and is associated with reduced fertility outcomes.

In mice, aged epididymides exhibit tubular and cellular degeneration and accumulate structurally abnormal sperm with reduced motility (Lopez-Trinidad et al., 2021). Additionally, the epididymis of 24-month-old mice shows accumulation of DNA damage and intense β-galactosidase staining, a commonly used biomarker for cellular senescence in both *in vivo* and *in vitro* studies (Lopez-Trinidad *et al*., 2021). Notably, the antioxidant capacity of the epididymis declines with aging (Noblanc et al., 2020).

## Male fertility decreases with age

Several studies have demonstrated that the compromised DNA integrity of aged sperm adversely affects fertilization and the normal development of embryos. Male mice older than 18 months exhibit smaller litter sizes and reduced pup numbers per vaginal plug (Endo et al., 2024). Interestingly, pregnancy rates in females with vaginal plugs were not influenced by male age, indicating that reduced litter size is a primary indicator of diminished *in vivo* fertility in aged males (Endo *et al*., 2024). In humans, increasing paternal age has been shown to have detrimental effects on the time to pregnancy (TTP). Retrospective studies and meta-analyses have found that the probability of achieving pregnancy within 6 or 12 months is lower for men over 40 years of age (Ford et al., 2000; Priskorn et al., 2014; Eisenberg and Meldrum, 2017; Lindahl-Jacobsen et al., 2023), a finding independent of the female partner’s age (Hassan and Killick, 2003).

APA is also a critical consideration in *in vitro* fertilization (IVF) procedures. High rates of sperm DNA fragmentation, commonly observed in older fathers, significantly reduce the success rates of fresh embryo transfer cycles, as well as the clinical pregnancy rates in frozen embryo transfer cycles (Virro et al., 2004; Dar et al., 2013; Gunes et al., 2016; Zhu et al., 2022; Li et al., 2024).

## Cellular and environmental factors associated with impaired spermatogenesis in advanced paternal age

APA is unequivocally linked to a range of adverse reproductive outcomes. Various factors can accelerate reproductive aging in males by impairing sperm quality, testicular function, and overall fertility. These factors often destabilize the genome and epigenome of germ cells, exacerbating the natural decline in male reproductive health associated with aging. Key contributors to male reproductive aging and its consequences include oxidative stress, replication errors and cumulative environmental exposures. 

### Oxidative stress during aging in germ cells

Aging in the male germ line is associated with a reduction in antioxidant capacity and an increase in intracellular ROS levels, resulting in oxidative stress (OS). This imbalance has been documented in the male reproductive tract (Sikka, 2001), as well as in spermatocytes, round spermatids (Selvaratnam et al., 2015), mature spermatozoa (Ozkosem et al., 2015), and Leydig cells (Luo et al., 2006), all of which are linked to lower-quality gametes and fertility issues.

Age-related OS particularly affects the function and genomic stability of male gametes. Men over 40 years old exhibit elevated ROS levels in their seminal fluid, which have been associated with reductions in sperm concentration and motility (Cocuzza et al., 2008). Additionally, aged sperm are more susceptible to chromatin packaging alterations, which increases their sensitivity to OS conditions (Zubkova and Robaire, 2006; Ozkosem et al., 2015). The detrimental effects of ROS on aged sperm also include lipid peroxidation, DNA fragmentation, and telomere shortening (Bui et al., 2018).

### Replication errors

DNA replication is an exceptionally precise process. During each replication cycle, replicative polymerases copy over 3 × 10⁹ bases with a mutation rate ranging from 10⁻⁹ to 10⁻¹⁰ (Drake et al., 1998; Saini et al., 2016; Kuchakulla et al., 2021). However, low-fidelity polymerases and DNA strand slippage can cause nucleotide insertions or deletions, leading to replication errors and the occurrence of *de novo* mutations (Kuchakulla *et al*., 2021). DNA replication errors are commonly observed in spermatogonial stem cells. In a 20-year-old man, sperm production involves approximately 150 spermatogonial divisions, whereas in a 50-year-old man, sperm production results from around 840 SSC divisions. (Sharma et al., 2015). Such replication errors contribute to the phenomenon of selfish spermatogonial selection. In so doing, replication errors in SSCs contribute to the alterations of the mutational profile of sperm as men age and to the increased accumulation of de novo mutations in the offspring of older fathers.

### Environmental exposures during lifetime

Aging is significantly influenced by environmental and individual lifestyle factors. The male germ line is particularly sensitive to pollutants found in air, water, and soil; plastic-derived compounds (such as bisphenol-A (BPA), bis(2-ethylhexyl)phthalate (DEHP), and dibutyl phthalate (DBP)); endocrine disruptors; drugs; diet; body weight; and metabolic diseases (Table 1). These lifestyle and environmental exposures can alter the genome and epigenome of germ cells, potentially leading to mutations in gametes that may be transmitted to the offspring (Linschooten et al., 2013) 

**Table 1-:** Impact of environmental exposures on male reproductive function and their consequences for offspring (hormonal causes are not included).

Environmental exposure	Effect in male reproductive health	Effect in the offspring	Main mechanisms of action	References
Air pollutant	Reduced sperm motility, sperm concentration, motility. Altered sperm morphology. May causes sperm DNA damage and fragmentation Altered DNA methylation 5-hmC levels	Not reported	Increase in reactive oxygen species (ROS)	Fathi Najafi et al., 2015; Deng et al., 2016; Kumar et al., 2021; Cheng et al., 2022; Omolaoye et al., 2024
Endocrine disruptors (includes plastic-derived compounds)	Reduced sperm count, volume, motility, morphology, and concentration	Not reported	Increase in reactive oxygen species (ROS) Activation of PPARα and PPARγ pathways Disrupted tight junctions between Sertoli cells and spermatogenic cells	Rehman et al., 2018
High fat diet	Low sperm count, viability, and motility Abnormal sperm morphology Transcriptional changes and increased N6-Methyladenosine Methylation	Increased body weight, liver and epididymal white adipose tissue (eWAT) weights Increased liver triglyceride content Upregulation of genes involved in lipid and triglyceride metabolism (liver and eWAT) Impaired pancreatic β-cell function in female offspring Metabolic and reproductive disorders in male offspring	Increase in reactive oxygen species (ROS)	Valenzuela et al., 2012; Aizawa et al., 2022; Sun et al., 2023
Heat stress	Decreased sperm concentration, sperm count, motility Abnormal sperm morphology Spermatogenic arrest Germ cell death Sperm DNA damage Altered testicular gene expression Sub and infertility	Not resolved	Decreased antioxidant capacity and enzymatic antioxidants Increase in reactive oxygen species (ROS) Imbalance in the testicular microenvironment (increase in *Asticcacaulis* spp.) Disturbed retinol metabolism Chromatin remodelling failure during spermiogenesis	Palmer et al., 2012; Grandjean et al., 2015; Craig et al., 2017; Liu and Ding, 2017; Barbagallo et al., 2021; George et al., 2023
Sedentary lifestyle	Sperm nuclear DNA damage and fragmentation	Impaired glucose tolerance in male offspring Weight gain in the male offspring	Increase in reactive oxygen species (ROS) Chromatin remodelling failure during spermiogenesis	Eisenberg et al., 2015; Stanford et al., 2018; Foucaut et al., 2019; Gill et al., 2019; Hart and Tadros, 2019; Balawender and Orkisz, 2020; Batista et al., 2020
Obesity	Decreased sperm concentration, sperm count, motility Abnormal sperm morphology DNA fragmentation Reduced sperm binding to oocyte and fertilization rates. Changes to epigenetic modifiers Altered DNA methylation pattern in sperm	Reduced implantation, blastocyst development,and live birth rates Altered behavior and glucose metabolism	Increase in reactive oxygen species (ROS)	Palmer et al., 2012; Grandjean et al., 2015; Katib 2015; Potabattula et al., 2019; George et al., 2023
Psychological stress (high cortisol levels)	Apoptosis of germ cells and Leydig cells Decreased sperm concentration and motility Abnormal sperm morphology Alterations in sperm long RNA	Risk-taking behaviours Depression Increased food intake Sensitivity to insulin challenge Metabolic and hepatic disorders	Increase in reactive oxygen species (ROS)	Eskiocak et al., 2006; Wu et al., 2016; Wdowiak et al., 2017; Ilacqua et al., 2018; Gapp et al., 2020; Braverman et al., 2024
Recreational drugs and chemicals (Tobacco, cannabis, alcohol)	Sperm hyperactivation Decreased semen volume, sperm count and concentration Differential miRNA expression in sperm DNA damage Altered DNA methylation pattern in sperm	Increased body fat Long-term metabolic syndrome Altered methylation of the DLK1 DMR locus Altered neurodevelopment	Increase in reactive oxygen species (ROS) DNA adducts formed by Benzo(a)pyrene (tobacco)	Sharma et al., 2004; Talebi et al., 2011; Marczylo et al., 2012; Taha et al., 2012; Dai et al., 2015; Payne et al., 2019; Schrott et al., 2020; Slotkin et al., 2020; Srinivasan et al., 2021; Liu et al., 2022

The mechanisms through which environmental factors impair male reproduction are diverse, yet many converge on common pathways, including oxidative stress, DNA damage, and epigenetic alterations in germ cells and sperm. Certain factors, such as toxins and microplastics, can induce epigenetic mutations (epimutations) and modify non-coding RNAs (ncRNAs), leading to phenotypic changes that may be transmitted across generations. (Hart and Tadros, 2019; Cescon et al., 2020; Yin et al., 2022). 

Oxidative stress is the primary mechanism of damage induced by environmental agents, acting as a unifying factor for pollutants in air, soil, and water, as well as for endocrine disruptors, chemicals, and physical agents (Rehman et al., 2018; Kumar et al., 2021; Munzel et al., 2023). Additionally, lifestyle habits such as chronic stress, poor diet, drug abuse, and a sedentary lifestyle also induce oxidative stress and genomic alterations, posing significant risks to the male reproductive system and the health of future offspring (Table 1) (Craig et al., 2017; Salas-Huetos et al., 2017; Ilacqua et al., 2018; Gill et al., 2019; Srinivasan et al., 2021; da Silva et al., 2022; Liu et al., 2022; Braverman et al., 2024).

## DNA Damage and genome instability: Central mechanisms of male aging phenotypes

Although the causes of male reproductive aging are multifactorial, the phenotype observed in the testes and spermatogenesis, as well as the factors that accelerate reproductive aging, share a common feature: the induction of DNA damage and the generation of genome instability. DNA damage thus influences on all major aspects of the ageing phenotype. Some of the physiological alterations caused by DNA damage in turn boost genome instability in germ cells, thereby amplifying the deterioration of reproductive homeostasis during ageing. The strong mechanistic link between DNA damage and ageing, and the role of DNA and chromatin as the primary templates for all cellular functions, suggest DNA damage as a candidate for the primary cause of male reproductive ageing. The cellular and physiological changes resulting from DNA damage in germ cells accelerate the decline of reproductive homeostasis during aging as well as the generation of pathogenic mutations in the offspring associated with the risk to develop disorders

Paternally derived mutations are estimated to account for approximately 75% of all *de novo* mutations in humans, occurring at a rate 6-9 times higher than those originating from maternal germ cells (Aitken and Lewis, 2023). Notably, the incidence of DNA mutations, such as single nucleotide polymorphisms (SNPs), is strongly correlated with paternal age at the time of conception, with an increase of two mutations per year of the father’s age (Kong et al., 2012). This mutation rate is significantly higher than the rate observed in females (Kong *et al*., 2012). Consequently, the male germline is the primary source of *de novo* mutations in offspring, a process that is exacerbated by advanced paternal age (Bergero et al., 2021). As previously mentioned, the male germline is particularly susceptible to oxidative stress, leading to DNA damage (Figure 2). Although the specific molecular mechanisms driving the aging phenotype in germ cells remain poorly understood, the role of DNA damage and genome instability is unequivocal.

DNA damage occurs regularly in mammalian cells due to exposure to both external and internal genotoxic agents. This damage includes single-strand breaks (SSBs), double-strand breaks (DSBs), mismatches, base substitutions, and inter- or intrastrand cross-links. While most of these lesions are repaired by specialized DNA repair mechanisms, some remain unresolved or are inaccurately repaired, leading to cumulative genomic damage over time (Schumacher et al., 2021). As individuals age, the efficiency of DNA repair mechanisms declines, resulting in increased DNA mutations and genomic instability (Figure 2) (Gorbunova et al., 2007). In the context of male reproductive aging, this accumulation of unrepaired DNA damage significantly impacts the germline. The progressive decline in DNA repair capacity with age leads to a buildup of mutations in male germ cells. This genomic instability not only leads to the deterioration of germ cells and sperm quality but also increases the risk of transmitting *de novo* mutations to offspring, a hallmark of male reproductive aging. Consequently, aging in males is closely linked to decreased fertility, compromised sperm integrity, and an elevated risk of passing on genetic disorders to future generations.

### Defects in DNA repair and DNA damage response in spermatogonial stem cells

In germ cells, two key forms of DNA damage and their associated repair pathways are notably affected by male aging. The first is the diminished removal of aberrant DNA bases induced by reactive oxygen species (ROS), a result of the reduced activity and efficiency of the base excision repair (BER) pathway (Paul et al., 2011; Zhao et al., 2021). One notorious example is the accumulation of 8-oxo-2′-deoxyguanosine (8-oxo-dG), a common and mutagenic lesion caused by oxidative stress, which is significantly elevated in aged rats and mice (Cao et al., 2004; Zhao *et al*., 2017).

Considerable attention has been given to the impairment of the BER pathway in spermatogonia and spermatocytes. Normally, spermatogonial stem cells exhibit low levels of spontaneous mutation; but this protection is substantially compromised with aging. Spermatogonia from older fathers are strongly associated with the appearance of *de novo* mutations due to the cumulative effects of DNA replication errors and defective DNA repair in a ROS-enriched environment (Figure 2) (Pohl et al., 2021; Aitken, 2022).

Replication errors in spermatogonia frequently occur at hypermutable hotspots in the genome, leading to recurrent mutations such as the FGFR3 gene (c.1138G>A, p.G380R), FGFR2 gene substitutions (c.755C>G, p.S308W and c.758C>G, p.P253R), RET gene substitution (c.2943T>C, p.M918T), HRAS (c.34G>A ) and a PTPN11 mutation (c.922A>G, p.N308D) (Arnheim and Calabrese 2016). These mutations contribute to the high incidence of autosomal dominant paternal age effect (PAE) disorders in the offspring, such as achondroplasia. Apert syndrome thanatophoric dysplasia, Costello syndrome and Pfeiffer’s syndromes (Figure 2) (Aitken, 2022)

However, replication errors alone cannot fully explain the elevated rate of *de novo* mutations in aged germ cells (Cioppi et al., 2019). Increased ROS levels and reduced BER pathway activity in aging further contribute to the heightened background mutation rate in spermatogonia (Nguyen-Powanda and Robaire, 2021; Aitken and Lewis, 2023).

Mutations in spermatogonial stem cells can trigger apoptosis or affect genes involved in the tyrosine kinase pathway, which regulates the balance between proliferation and differentiation. This disruption may lead to increased cell proliferation, consistent with the selfish spermatogonial selection model (Dong et al., 2022). The interplay of these mechanisms likely explains the paternal age effect on spermatogonia, as well as the susceptibility of the entire spermatogenic cycle to mutagenic exposures.

### Age-related defects in the DNA damage response in spermatocytes

Age-related deficiency in the BER pathway, as well as in homologous recombination (HR) and non-homologous end-joining (NHEJ), have also been described in meiotic spermatocytes. During meiosis, spermatocytes experience significant genomic changes, making their genome particularly vulnerable. Programmed DNA double-strand breaks (DSBs) occur in early meiotic spermatocytes to facilitate the proper pairing and synapsis of homologous chromosomes. These DSBs are primarily repaired through homologous recombination (HR), with a subset resolved as crossovers (CO), which are essential for accurate chromosome segregation. Meiosis is also marked by extensive changes in gene expression and chromatin structure, with many transcripts required for spermiogenesis, fertility, and embryogenesis transcribed during the pachytene stage. Consequently, precise DNA repair during meiosis is crucial for generating genetically and epigenetically normal sperm and ensuring successful embryogenesis.

In rodents, aging is associated with increased oxidative stress, leading to elevated levels of 8-oxo-dG and lipid peroxidation, as well as alterations in key DNA repair pathways, including base excision repair (BER), nucleotide excision repair (NER), and homologous recombination (HR) in spermatocytes (Paul et al., 2011; Nguyen-Powanda and Robaire, 2021). In aged rats, significant changes were observed in the NER and BER pathways, particularly in genes like *Ercc1*, *Xpa*, *Rpa1*, and *Ercc4 (Xpf)*, though not at the protein level. Notably, 30% of BER genes were affected in aged spermatocytes, with *Ogg1* upregulated, and *Ape1*, *Fen1*, and *Xrcc1* downregulated, which was also reflected in lower protein levels of APE1, XRCC1, and FEN1 (Paul *et al*., 2011). This defective BER pathway led to increased 8-oxo-dG in sperm (Paul *et al*., 2011), suggesting that DNA damage sustained during spermatogenesis is transmitted to sperm.

Aging is also associated with significantly elevated levels of double-strand breaks (DSBs) in spermatocytes (Figure 2), likely impairing the homologous recombination (HR) and non-homologous end joining (NHEJ) pathways. In aged mouse spermatocytes, there is a marked increase in DNA damage, accompanied by a decline in the expression of key DNA repair genes, including *Brca1*, *Atm*, *Mre11*, *Dmc1*, and *Rad51* (Stobezki et al., 2020). Similarly, aged *Sod1-/-* mice exhibit reduced transcription and immunostaining of *Rad51* and *LigIV*, two essential proteins in the HR and NHEJ pathways, respectively (Nguyen-Powanda and Robaire, 2021). The formation and regulation of crossovers (COs) during meiosis are critical for maintaining genomic stability, and several HR proteins, including *Rad51*, play a central role in facilitating meiotic recombination (Lao et al., 2013).

In aged mice, spermatocytes demonstrate increased levels of recombination between homologous chromosomes (Vrooman et al., 2014). However, despite the essential role of COs in establishing physical connections between chromosomes to ensure their correct segregation, aging is associated with increased frequencies of unpaired homologs, particularly involving the X and Y chromosomes during Prophase I, as well as univalent autosomes and sex chromosomes in Metaphase I (Vrooman *et al*., 2014). When interhomolog recombination is compromised, it has been suggested that the system compensates by increasing crossover events and inducing additional DSBs in response to defective homolog interactions (Figure 2) (Lao et al., 2013). While these mechanisms may maintain crossover homeostasis, the precise meiotic consequences of altering the proteins involved in the initiation and maturation of recombination remain unknown.

Aged mice also exhibit a reduction in the expression of the spindle assembly checkpoint protein *BubR1* in the testes, which suggests that the proper segregation of homologous chromosomes during meiotic metaphase I and anaphase I may be impaired with age (Baker et al., 2004). However, no evidence of aneuploidy is observed in metaphase II, indicating that meiotic checkpoints eliminate spermatocytes with chromosomal errors in aged males (Vrooman et al., 2014). Because recombination foci are present on all chromosomes during Prophase I in aged spermatocytes, it has been hypothesized that most autosomal univalents at Metaphase I result from premature cohesion loss (Vrooman *et al*., 2014). It would be interesting to investigate whether mutations in cohesion genes arise in aged spermatogonia, as such mutations could potentially disrupt chromosome segregation during metaphase I. Furthermore, given the current data, it is possible that the formed COs are less efficient at facilitating recombination and establishing effective physical interactions between chromosomes. This raises the critical question of whether defective DNA damage repair pathways compromise normal meiotic processes, leading to a more error-prone genomic DNA damage response (DDR) (Figure 2).

The strict checkpoint mechanisms during male meiosis, along with induced apoptosis, likely account for the severe effects of aging on spermatocytes in the seminiferous tubules (Figure 1). However, spermatocytes with mutations can still complete meiosis and differentiate into spermatids (Manterola et al., 2009), suggesting the existence of a threshold beyond which genomic damage is not detected by meiotic checkpoints and instead is passed on to spermatids (Figure 2). This DNA damage tolerance mechanism has been well-documented in somatic cells, where it allows DNA replication to proceed despite lesions, leaving damage to be repaired at a later stage (Cesare et al., 2013; Ghosal and Chen, 2013). Similarly, in meiotic cells, it is plausible that in response to alterations in canonical DDR pathways, alternative signaling mechanisms are activated to repair the damaged DNA. However, these non-canonical pathways may also result in *de novo* mutations or epigenetic changes, potentially contributing to the paternal age effect (PAE) disorders observed in offspring APA individuals.

### Age-related defects in DNA damage response in spermatids and sperm

Unlike spermatogonia and spermatocytes, spermatids are haploid cells, and their DNA damage is primarily repaired through classical or alternative non-homologous end joining (NHEJ) pathways (Ahmed et al., 2015). In aging mouse spermatids, oxidative stress is associated with increased lipid peroxidation and elevated levels of 8-oxodG, leading to higher levels of double-strand breaks (DSBs) (Nguyen-Powanda and Robaire 2021). Due to limited DNA repair capacity in spermatids, defects in repair and the persistence of DSBs can result in elevated sperm DNA damage and fragmentation (Figure 2), contributing to the subfertility commonly observed in APA (Aitken and De Iuliis 2007). Spermatids are also particularly vulnerable to DNA damage because they express a unique set of genes crucial for gametogenesis, fertility, and embryonic development (Pandey et al., 2019). During spermatid elongation, chromatin undergoes significant remodeling and genome condensation, replacing histones with protamines to form mature sperm. Altered DNA protamination has been observed in spermatids. Elderly subjects showed a notable reduction in the expression of PRM1 and PRM2 (Figure 2), while there were no significant changes in the expression levels of TNP1 or TNP2 compared to young men (Paoli et al., 2019). This may be linked to the increased sperm DNA damage observed in elderly subjects, suggesting a significant vulnerability in chromatin integrity (Sharma et al., 2015; Paoli *et al*., 2019). Further research is required to elucidate how aging affects the mechanisms involved in the histone-to-PRM transition through changes in epigenetic modifications, ultimately leading to decreased male fertility.

Another source of DNA damage in spermatids is the persistence of lesions from meiosis that remain unrepaired as spermatids transition through DNA repair-competent stages of spermatogenesis (Ahmed et al., 2015; Marchetti et al., 2015). DNA damage in sperm has been extensively studied and is often characterized by DNA fragmentation and abnormal packaging (Sharma et al., 2015). Such defects may arise from persistent genomic instability introduced earlier in germ cell development. Additionally, reduced protection against oxidative stress during sperm maturation in the epididymis may further contribute to the poor genomic integrity of aged sperm (Noblanc et al., 2020; Wu et al., 2020).

DNA damage can accumulate during spermatogenesis, persist in the sperm, and be transmitted to the zygote (Figure 2). Although the oocyte typically repairs sperm-derived genomic damage, its capacity for DNA repair is limited, especially in aged oocytes. This limitation can lead to misrepair by the oocyte’s DNA repair machinery, increasing the likelihood of inheriting genomic mutations in the zygote (Marchetti et al., 2015; Aitken, 2022). Furthermore, the effects of paternal age are amplified when the maternal age exceeds 35 years (Fisch et al., 2003).

Taking together, these observations highlight the critical role of DNA damage and repair during spermatogenesis as driving factors in male reproductive aging and the inheritance of genomic instability in offspring.

## Integrating environmental factors and DNA Damage: epigenetic alterations in aged germ cells

Epigenetic alterations are key features of aging, influencing genome function through mechanisms like DNA methylation, histone modifications, chromatin remodeling, and non-coding RNA transcription. In germ cells, these epigenetic regulators create specific chromatin codes that control the genomic organization and activity of spermatogonia, spermatocytes, spermatids, and sperm. Many of the epigenetic marks formed during spermatogenesis are passed to the oocyte during fertilization, contributing to the genomic reprogramming of the embryo. 

Several studies have addressed that aging has significant effects on the epigenetic programming of the sperm. Changes in global DNA methylation patterns with paternal age contribute to genomic instability, epigenetic mutations, and an increased risk of transmitting developmental disorders to offspring (Figure 2). MethylC-capture and pyrosequencing analysis of sperm from men aged >46 years old identified thousand age-related CpG sites with significant differential methylation (Figure 2) (Jenkins et al., 2013; Jenkins *et al*., 2014; Cao et al., 2020). Among t798 differentially methylated regions (DMRs), 62% were hypermethylated and 38% were hypomethylated (Cao *et al*., 2020). Hypomethylated regions with high CpG density were found to drive nucleosome retention and were frequently located near transcription start sites (TSS), while hypermethylated CpG sites were more commonly situated in distal regions of genes (Cao *et al*., 2020). Age-associated hypomethylated CpG clusters were particularly dense on chromosome 4, overlapping with the PGC1α locus, a protein involved in metabolic aging, and on chromosome 16, overlapping with the RBFOX1 locus, a gene linked to neurodevelopmental disorders (Cao *et al*., 2020). Hypomethylation at REST/NRSF binding motifs in aged sperm leads to the upregulation of REST/NRSF target genes in the embryonic forebrain (Yoshizaki et al., 2021).

The DMRs in aged sperm were enriched in genes related to development, neuron projection, differentiation, recognition, and behavior, and were also associated with conditions like bipolar disorder and schizophrenia (Jenkins et al., 2014; Jenkins *et al*., 2018; Cao et al., 2020; Yoshizaki et al., 2021), influencing neurodevelopmental pathways in the offspring of older fathers. Furthermore, age-related hypomethylation was correlated with H3K4 and H3K27 methylation (Jenkins *et al*., 2014), underscoring the intricate relationship between DNA methylation changes and histone modifications in aged sperm.

Age-related changes in sperm DNA methylation have been shown to negatively impact the outcomes of intracytoplasmic sperm injection (ICSI) and reduce fertilization rates (Mohammed and Maged 2023). Notably, the levels of 5-methylcytosine (5-mC) and its intermediate form, 5-hydroxymethylcytosine (5-hmC), increase by 1.76% and approximately 5% per year of life, respectively, in men. These increases contribute to gene silencing, underscoring the epigenetic risks associated with APA in the programming of the embryo (Jenkins et al., 2013). Aging also results in an increase in 5hmC levels in gene bodies, accompanied by a corresponding decrease in 5mC levels within these regions (Liao et al., 2021). These findings suggest that APA-related effects in offspring arise not only from DNA mutations but also from epigenetic changes. Age-related alterations in the epigenetic landscape increase the susceptibility of offspring to diseases, a risk that can persist for multiple generations. Importantly, age-related DNA methylation changes were particularly enriched in subtelomeric regions, which may evade large-scale epigenetic reprogramming events in the embryo (Guibert et al., 2012). Consequently, epigenetic mutations occurring in germ cells can be transmitted to the offspring, contributing to paternal age effect (PAE) disorders commonly observed in children of older fathers. Furthermore, histone modification analyses in aged spermatogonia have shown reduced levels of H3K27me3 in pro-differentiation genes that are essential for spermatogonial stem cell development, along with aberrant distribution of this histone mark in genes involved in the Wnt and TGF-β signaling pathways (Liao *et al*., 2021). These changes highlight the intricate relationship between aging, epigenetic modifications, and sperm.

## The interplay between epigenetics and DDR as possible sources of genome instability during aging

Epigenetic alterations in aging are closely linked to DNA damage and deficiencies in the DNA damage response (DDR). Aging-induced changes in the epigenetic landscape impact the initiation, activity, and resolution of DDR processes, as well as the selection of DNA repair pathways (Loizou et al., 2006; Soto-Palma et al., 2022). One hallmark of aging is the significant accumulation of the histone variant H2AX phosphorylated at serine 139 (γH2AX) within the genome. This accumulation is associated with the persistent activation of DDR signals and elevated expression of γH2AX during aging (Downey and Durocher, 2006). Epigenetic modifications can also directly contribute to DNA damage and *de novo* mutations. For instance, the deamination of 5mC into thymine results in a mismatch, which is typically repaired by the base excision repair (BER) pathway to restore a G pair. However, in aging cells, the efficiency of the BER pathway declines, leading to an accumulation of C>T mutations, particularly in methylated CpG dinucleotides (Iengar, 2012). Additionally, thymine’s methyl group is prone to oxidation, forming 5-hydroxymethyluracil (5hmU), 5-formyluracil (5foU), and 5-carboxyuracil (5caU) (Iengar, 2012).

Changes in 5hmC, a product of 5mC, have been shown to cause a decline in spermatogonial stem cells and impair spermatogenesis, thereby accelerating age-related infertility (Huang et al., 2020). Several DNA methyltransferases (DNMTs) are directly involved in DNA damage repair, forming complexes with DNA repair proteins to modulate DDR signaling and reshape the chromatin environment surrounding DNA lesions (Jin and Robertson, 2013).

Spermatogenic cells are uniquely capable of switching between DNA repair pathways in response to persistent damage (Enguita-Marruedo et al., 2019). However, issues arise when distinct patterns of DNA methylation and histone modifications are established during alternative DDR processes and subsequently transferred between cells (Wajed et al., 2001). This transfer of aberrant chromatin marks can lead to the emergence of epigenetic mutations. If a defective DNA repair pathway is activated to address these epigenetic alterations, *de novo* DNA mutations may occur in germ cells, further contributing to genomic instability.

## Aging alters the transcriptional program of male testicular somatic and germ cells, contributing to reproductive dysfunction and heritable changes in offspring

In addition to the genomic and epigenomic alterations in male germ cells associated with aging, several studies have shown that aging also induces transcriptomic changes in germ cells and the epididymis, affecting both mRNAs and non-coding RNAs (ncRNAs), including miRNAs.

RNA-seq analysis of aged testes and epididymides reveals that Sertoli cells show upregulation of genes related to inflammation and cellular senescence, particularly senescence-associated secretory phenotype (SASP) factors that promote inflammation (Lopez-Trinidad et al., 2021; Endo et al., 2024). Aged testes also exhibited a down-regulation of genes involved in DNA repair and telomere maintenance across Sertoli, Leydig, and germ cells (Zhang et al., 2023). The expression of the Wilms’ Tumor 1 (*WT1*) gene plays a crucial role in preventing cellular senescence in Sertoli cells and maintaining a supportive microenvironment for germ cells. In aging, a significant downregulation of *WT1* has been observed, which leads to increased cellular senescence and disruption of tight junctions in Sertoli cells (Huang et al., 2023). This impaired cellular function is believed to contribute to the formation of a harmful microenvironment for spermatogenesis, resulting in the reduced sperm production and compromised spermatogenesis commonly associated with aging (Huang *et al*., 2023).

Aged testes also show an altered gene expression of *Pick1* and *Fads2*, which are crucial for acrosome formation, and the transcription of protamine genes *Prm1* and *Prm2* (Sharma et al., 2015). Other downregulated genes in the aged testis include *PolB*, encoding a DNA polymerase involved in the BER pathway, which may explain DNA mutations and genomic instability associated with APA; *Nrg1*, encoding Neuregulin 1, essential for the proliferation and differentiation of spermatogonia, Leydig cells, and Sertoli cells; *Tet1*, encoding Ten-eleven translocation 1, vital for cytosine demethylation; and *Odf2*, *Akap4*, *Ift74*, *Carma*, and *Crisp2*, which play roles in sperm differentiation and tail development (Huang et al., 2023). Intriguingly, among all testicular cells telocytes demonstrated the most significant alterations in gene expression during testicular aging (Guo et al., 2024). Remarkably, while somatic cells exhibited a higher number of age-related differentially expressed genes, germ cells showed higher age-related alternative splicing changes (Guo *et al*., 2024), highlighting distinct molecular aging patterns between the cell types in the testis.

Age induced ROS also induce changes in small non-coding RNA profiles in sperm, particularly transfer RNA-derived small RNAs (tsRNAs) and ribosomal RNA-derived small RNAs (rsRNAs). Oxidative stress-induced tsRNAs and rsRNAs in sperm have been linked to depressive-like and anxiety-like behaviors in offspring (Ren et al., 2022). Age related differentially expressed microRNAs in sperm were linked to signaling pathways previously implicated in aging, such as cellular senescence, insulin signaling, and regulation of cell metabolism, growth, proliferation and survival (Santiago et al., 2023). These findings suggest that age-related changes in microRNA regulation could contribute to the reduced fertility and adverse reproductive outcomes often observed in APA.

## Impact of advanced paternal age on offspring health: What is being inherited?

APA is increasingly recognized as a significant factor influencing both offspring health and the transmission of genetic and epigenetic traits. As previously mentioned, during aging genomic and epigenomic alterations in germ cells and sperm occur, with lifestyle and environmental factors further exacerbating these changes. These alterations lead to *de novo* DNA mutations, epimutations, and changes in RNA profiles in sperm, resulting in the reprogramming of various loci that are subsequently inherited by the offspring. That is, the dynamic of germ cells and sperm during aging, along with the lifestyle produce phenotypic changes in the offspring that may persist across multiple generations.

What, then, are the consequences of the genomic and epigenomic mutations arising in aged germ cells, carried by sperm, and further influenced by environmental and lifestyle factors? The initial effects of male aging in the offspring become evident during pregnancy and in newborns. APA has been linked to a range of adverse pregnancy outcomes and neonatal complications. APA is associated with an increased risk of miscarriage, abortion (Slama et al., 2003), poor embryo quality, stillbirth (Astolfi et al., 2004), low birth weight, fetal growth restriction, and the need for admission to a neonatal intensive care unit (Hurley and DeFranco, 2017). Additional risks include seizures and reduced life expectancy in the newborn (Wiener-Megnazi et al., 2012; Zhang et al., 2022). APA has also been implicated in maternal complications during pregnancy. Female partners of men over 45 years of age exhibit a higher incidence of hypertension and placental abnormalities, such as placental abruption and placenta previa (Alio et al., 2012). Abnormal genetic and epigenetic patterns in sperm can lead to embryonic failure and infertility, potentially playing key roles in cases of recurrent spontaneous abortion by affecting embryo implantation, growth and development (Zhou et al., 2021; Ma et al., 2023).

The increased DNA Damage and *de novo* DNA mutations and epimutations in aged sperm contribute to an increased risk of genetic disorders and developmental issues in offspring. As paternal age increases, there is a rise in T>G substitutions and a reduction in non-CpG C>T substitutions in the offspring, as well as several changes in the DNA methylation and histone modification patterns (Goldmann et al., 2019; Cao et al., 2020). Indeed, the increment in sperm chromatin abnormalities and DNA damage in aging men is becoming to be accepted as an explanation for the decline in fertility and paternal age effect disorders (PAE disorders) observed in older men (Wyrobek et al., 2006).

### Genetic disorders associated to advanced paternal age

Paternal age effect (PAE) disorders encompass genetic conditions likely caused by chromosomal abnormalities and *de novo* mutations in germ cells. These disorders include, at least, mutations in SSCs that are transmitted to sperm, such as single nucleotide substitutions in genes like FGFR2 and FGFR3 (Figure 2), which confer a selective advantage to mutated spermatogonia (Goriely and Wilkie, 2012). Additionally, gain-of-function mutations in genes associated with the RAS/MAPK signaling pathways have been implicated (Maher et al., 2014). Single nucleotide substitutions in RET genes are responsible for a range of autosomal dominant syndromes, including Pfeiffer syndrome, Crouzon syndrome, Apert syndrome, achondroplasia, thanatophoric dysplasia, and multiple endocrine neoplasia types 2A and 2B (MEN2A and MEN2B) (Orioli et al., 1995; Kovac et al., 2013). Notably, mutations in the FGFR2, FGFR3, and RET genes present in offspring are exclusively of paternal origin, and their occurrence increases with paternal age (Crow, 2000). Other conditions, such as Neurofibromatosis type I and Osteogenesis imperfecta, have also been linked to advanced paternal age (Carothers et al., 1986; Risch et al., 1987; Orioli *et al*., 1995). 

### Congenital abnormalities in offspring are linked to male reproductive aging.

The association between advanced paternal age and an elevated risk of congenital anomalies has been documented for many years (Figure 2). An elevated risk of neural tube defects, congenital cataracts, upper limb reduction defects, oral cleft, chondrodystrophy and Down syndrome has been observed with increasing paternal age (Polednak, 1976; McIntosh et al., 1995; Herkrath et al., 2012; Nybo Andersen and Urhoj, 2017). Additionally, there is a consistent pattern of paternal age-related risk for heart malformations such as ventricular and atrial septal defects and ductus arteriosus (Joinau-Zoulovits *et al*., 2020). 

### Male reproductive aging is associated with an increased risk of cancer and metabolic diseases in the offspring. 

Cancer risk is also associated to APA´s offspring (Figure 2). A positive association was found between paternal age and the incidence of cancer in the female reproductive organs, respiratory system, lungs and intrathoracic organs (Sun et al., 2020), retinoblastoma (Moll et al., 1996; Dockerty et al., 2001; Yip et al., 2006), prostate cancer (Zhang et al., 1999) and breast cancer (Choi et al., 2005). Advanced parental age is strongly associated with an increased risk of childhood cancer. Children born to fathers aged over 35 exhibited a significantly increased risk of developing retinoblastoma, with a marked upward trend as paternal age advanced. Furthermore, advanced paternal age was linked to a higher likelihood of non-Hodgkin’s lymphoma and gonadal germ cell tumors in the offspring (Domingues et al., 2022). This trend was even observed in South America (Rios et al., 2018). Other childhood cancers associated to APA are astrocytoma, Wilm’s tumor, acute lymphoblastic leukemia and acute myeloblastic leukemia (AML) (Yip *et al*., 2006; Larfors et al., 2012). APA have no risk of these cancer in adulthood (Nybo Andersen and Urhoj, 2017).

Metabolic diseases like childhood diabetes 1 (Cardwell et al., 2005) and eating disorders (Javaras et al., 2017) have also been associated to APA.

### Advanced paternal age is associated to neurodevelopmental disorders in offspring

A key hallmark of the paternal aging effect on offspring is the increased incidence of neuropsychiatric disorders (Figure 2). The mechanisms underlying this association have been linked to DNA mutations and abnormal epigenetic reprogramming, which are significantly enriched in genes essential for neurological development in both sperm from older fathers and blastocysts.

Several studies have found that *de novo* mutations produced in aged male germ cells have significant roles in the etiology of disorders such as schizophrenia (Rasmussen, 2006; Lehrer et al., 2016; de Kluiver et al., 2017; Janecka et al., 2017; Weiser et al., 2020), bipolar disorders (Brown et al., 2013; Chudal et al., 2014; Polga et al., 2022), anxiety-like behavior (Guo et al., 2021), and spectrum disorders like autism (Hultman et al., 2011; Goriely and Wilkie 2012; McGrath et al., 2014; Greenberg et al., 2020). Fathers aged ~50 years old have a 2.7-fold increased risk of having an autistic offspring than children born from fathers <29 years old (Hultman *et al*., 2011), and the offspring of men >55 years old have 1.37 times more risk to develop bipolar disorder (Frans et al., 2008). The risk of the offspring to develop autism and schizophrenia is higher in aged fathers than in advanced maternal age (Kimura et al., 2018). DNA methylation experiments have shown a causal relationship between changes in sperm DNA methylation and the risk of autism and schizophrenia in children of APA fathers. That is, aged sperm DNA methylation alterations were found in the promoter or gene body regions of 117 genes, including those related to schizophrenia and bipolar disorder (Jenkins et al., 2014). Global DNA methylation patterns in the brains of offspring from older fathers were found to resemble those observed in aged sperm (Kimura *et al*., 2018). This indicates that DNA methylation changes established in sperm due to advanced paternal age can persist and potentially influence gene expression in the offspring. Moreover, age-related changes in sperm DNA methylation increase the risk of children developing autism spectrum disorder as well as schizophrenia 

## The threshold hypothesis of male reproductive aging phenotype: Shaping a healthy reproductive outcome in advanced paternal age

DNA damage and epigenetic changes are not randomly produced in the genome. There is a clear causal relationship in aging: DNA damage alters the epigenome, which, in turn, affects DNA repair efficiency. These genomic alterations occur in cells that undergo significant chromatin and transcriptomic changes to produce haploid cells from a diploid parental cell, leading to detrimental consequences for male reproductive health and the well-being of offspring. Although the effects of aging on the epigenome of germ cells remain under investigation, it is evident that genomic integrity and epigenetic mechanisms are critical drivers of the aging phenotype in male reproduction.

**Figure 3 -:**
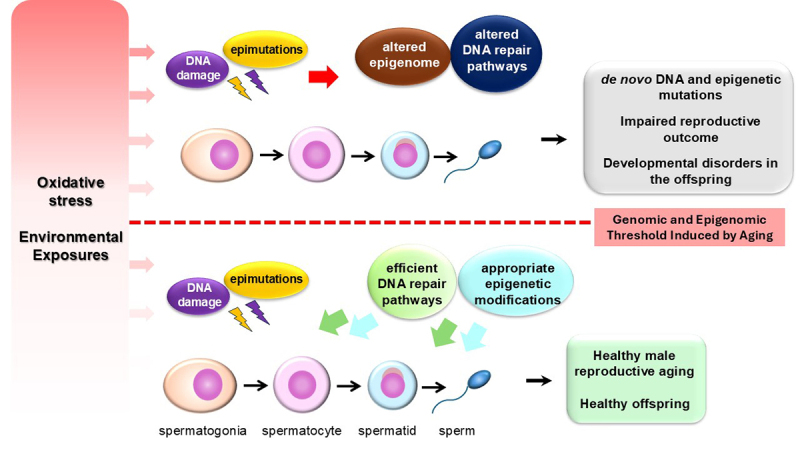
Genomic and epigenomic threshold induced by aging. Low-dose environmental exposures can elevate reactive oxygen species (ROS) levels, but the resulting DNA and epigenomic damage is typically addressed by homeostatic mechanisms (represented below the red dotted line). However, chronic or high-dose exposure overwhelms these repair systems, surpassing the genomic and epigenomic damage threshold and resulting in irreversible genetic and epigenetic mutations that destabilize the male germ cell genome (above the red dotted line). Aging impact in the genome of germ cells

A pertinent question arises: should aging be defined solely as a chronological factor, or should it be viewed as a biological phenomenon? As previously noted, various environmental factors contribute to the decline in male reproductive parameters, mirroring the effects of chronological aging. For instance, chromatin-modifying enzymes are susceptible to environmental agents, which can translate these influences into differential DNA methylation regions (DMRs) in both sperm and embryos (Ho et al., 2012; Donkin and Barres, 2018; Vozdova et al., 2022). These alterations accumulate over a lifetime, resulting in an age-acceleration pattern in sperm that shares the same genomic and epigenomic defects associated with advanced paternal age and paternal age effect disorders (Figure 3).

Given that cumulative environmental exposure is closely linked to chronological age, aging must be regarded as an intricate and multidimensional interplay of chronological, environmental, genetic, and epigenetic factors throughout an individual’s life, gradually affecting genomic and epigenomic stability. Consequently, male reproductive aging should not be perceived as a fixed concept but rather as one influenced by genetic and, primarily, environmental factors that result in significant variability in the epigenetic program and DNA damage response of germ cells (Figure 3). This variability is both sufficient and necessary to produce differences in the male reproductive aging phenotype among individuals within a population. In this context, the susceptibility to developing male reproductive defects hinges on a threshold of genomic and epigenomic integrity in germ cells. Low-dose exposures to harmful environmental factors can elevate ROS levels and affect DNA and the epigenome; however, homeostatic mechanisms-such as DNA repair, chromatin remodeling complexes, and antioxidant enzymes-typically resolve such damage. At this stage, any epigenetic alterations remain reversible, and normal genomic functions in germ cells are preserved. However, sustained high doses of harmful environmental factors, resulting from chronic exposure over a lifetime, can overwhelm and impair the activity and specificity of DNA damage repair proteins and chromatin remodeling complexes. Consequently, the reversibility of epigenetic marks is compromised, leading to genetic and epigenetic mutations that risk the stability and integrity of the male germ cell genome (Figure 3).

A father’s lifetime exposure to environmental factors can either compromise spermatogenesis and lead to reproductive issues and adverse offspring health or result in healthy reproductive outcomes, even with advanced paternal age, thus reducing disease risks for descendants. Positive lifestyle choices, such as consuming antioxidant-rich diets (e.g. polyphenols), exercising regularly, reducing gonadal heat stress, maintaining a healthy weight, avoiding harmful substances, and practicing meditation, have been shown to improve male reproductive health. These factors are linked to increased expression of antioxidant enzymes, normalization of sperm transcripts, enhanced levels of NAD+-dependent deacetylases, restoration of epigenetic modifications, decreased oxidative DNA damage, improved sperm motility and count, and reduced sperm DNA fragmentation (Ly et al., 2015; Dhawan et al., 2018; Ferramosca et al., 2021; Barbagallo et al., 2022; Minas et al., 2022; Qi et al., 2022; Abraham et al., 2023; Ajayi et al., 2023).. Overall, adopting a healthy lifestyle may help mitigate the effects of aging on germ cells and enhance male reproductive functions, benefiting the health of offspring.

In addition, various pharmacological and gene-targeting therapies have been explored for addressing male reproductive aging. Intranasal administration of nerve growth factors, medicinal herbs, and melatonin supplementation have been shown to improve the effects of oxidative stress on spermatogenesis and promote its restoration see (Dong et al., 2022).

Considering that advanced paternal age is a global public health concern, the reproductive and molecular aging effects on the male germ line must be factored into counseling interventions aimed at countering the premature aging of the reproductive system in men who delay fatherhood. This approach is vital for mitigating the harmful impact of APA on offspring health. A statement on genetic counseling for advanced paternal age has been published, emphasizing the need for all stakeholders to collaborate in disseminating knowledge about the adverse effects associated with APA (Toriello et al., 2008). Additionally, it is crucial to develop public policies and management strategies that address the urgent issue of APA in men’s reproductive health and its implications for health systems.

Unraveling the causal mechanisms underlying male reproductive aging holds great significance for the development of diagnostic, prognostic, and therapeutic tools designed to improve men’s reproductive health and reduce the risk of diseases in offspring associated with advanced paternal age.
